# Recording Characteristics, Microstructure, and Crystallization Kinetics of Ge/GeCu Recording Film Used for Write-Once Blu-Ray Disc

**DOI:** 10.3390/ma9110953

**Published:** 2016-11-23

**Authors:** Sin-Liang Ou, Feng-Min Lai, Wei-Kai Wang, Shih-Yung Huang, An-Cheng Sun, Ching-Ho Tien, Zhi-Jia Xu, Chin-Yen Yeh, Kuo-Sheng Kao

**Affiliations:** 1 Department of Materials Science and Engineering, Da-Yeh University, Changhua 515, Taiwan; slo@mail.dyu.edu.tw (S.-L.O.); fengmin@mail.dyu.edu.tw (F.-M.L.); wk@mail.dyu.edu.tw (W.-K.W.); s313326229@gmail.com (Z.-J.X.); 2 Department of Industrial Engineering and Management, Da-Yeh University, Changhua 515, Taiwan; syh@mail.dyu.edu.tw; 3 Department of Chemical Engineering and Materials Science, Yuan-Ze University, Chung-Li 320, Taiwan; acsun@saturn.yzu.edu.tw; 4 Department of Materials Science and Engineering, National Chung Hsing University, Taichung 402, Taiwan; jonath.tien@gmail.com; 5 CMC Magnetics Corporation, Taoyuan 333, Taiwan; JeremyYeh@cmcnet.com.tw; 6 Department of Computer and Communication, SHU-TE University, Kaohsiung 824, Taiwan

**Keywords:** Ge/GeCu bilayer, write-once blu-ray disc, microstructure, crystallization kinetic, jitter value

## Abstract

A Ge_67_Cu_33_ (16 nm) layer and a Ge (3 nm)/Ge_67_Cu_33_ (16 nm) bilayer were grown by sputtering at room temperature and used as the recording films for write-once blue laser media. In comparison to the crystallization temperature of Ge in a GeCu film (380.7 °C–405.1 °C), the crystallization temperature of Ge in a Ge/GeCu bilayer could be further decreased to 333.7 °C–382.8 °C. The activation energies of Ge crystallization were 3.51 eV ± 0.05 eV and 1.50 eV ± 0.04 eV for the GeCu and the Ge/GeCu films, respectively, indicating that the Ge/GeCu bilayer possesses a higher feasibility in high-speed optical recording applications. Moreover, the lower activation energy would lead to a larger grain size of Ge crystallization in the Ge/GeCu bilayer after the annealing process. Between the as-deposited and the annealed states, the optical contrasts (@ 405 nm) of the GeCu and the Ge/GeCu films were 26.0% and 47.5%, respectively. This reveals that the Ge/GeCu bilayer is more suitable for the recording film of a write-once blu-ray disc (BD-R) in comparison with the GeCu film. Based on the dynamic tests performed for 2× and 4× recording speeds, the optimum jitter values of the BD-R with the Ge/GeCu recording film were 7.4% at 6.3 mW and 7.6% at 8.6 mW, respectively.

## 1. Introduction

Recently, to prepare write-once blue laser media, various organic and inorganic materials have been used for the recording films. Of the inorganic materials, amorphous silicon (a-Si) is a promising candidate to serve as the optical recording film. However, its direct application in optical recording media is limited by the extremely high crystallization temperature (700 °C) of a-Si, meaning that a higher blue laser power would be required in the recording process. This will result in an increment in the cost of the blue laser pickup. Fortunately, the crystallization temperature of a-Si can be decreased efficiently via the metal-induced-crystallization (MIC) technique. Therefore, several metal/Si bilayers or metal–silicide alloy layers were presented to apply to the recording films of optical recording media [1,2,3,4,5,6]. On the other hand, physical and chemical properties of amorphous germanium (a-Ge) are similar to those of a-Si, which indicates that a-Ge also possesses great potential applications in optical recording media. Most importantly, compared with the high crystallization temperature of a-Si, a much lower one of 470 °C can be obtained in a-Ge. As a result, when the MIC method was used, a relatively low crystallization temperature (<400 °C) would usually be achieved. Up to now, various Ge-containing recording films, such as Ge/Au [7], Ni/Ge [8], GeCu [9], Ge/Cu [10], NiGe [11], and Ge/NiGe [12] were deposited as the recording layers of write-once blu-ray discs (BD-Rs). 

In the previous research, the Ge_100−*x*_Cu*_x_* films (*x* = 50–69) were deposited by co-sputtering of Ge and Cu targets [9]. The results demonstrated that the GeCu films possessed high optical contrast (at the wavelength of 405 nm), indicating that these films were suitable for BD-R applications. The crystallization mechanism and optical properties of the sputtered Ge/Cu bilayer was also investigated, revealing the possibility of its use as the recording film of BD-Rs [10]. However, in the above-mentioned studies, these two films combined with Ge and Cu materials were not employed for practical BD-R applications. Furthermore, in recent years, BD-Rs with Ge/metal-germanide (the Si/metal-silicide) recording films were proposed in our previous works [1,3,12]. The results showed that these BD-Rs had both higher optical contrast and the better recording performance than those with the metal/Ge or metal–germanide (the metal/Si or the metal-silicide) recording films. Up to now, the Ge/GeCu bilayer has yet to be applied in BD-Rs, and it can be expected that this recording film will be highly feasible for the fabrication of BD-Rs. 

In this study, we have proposed a Ge (3 nm)/Ge_67_Cu_33_ (16 nm) recording film to prepare the BD-R. The thermal properties, crystallization behavior, optical characteristics, and recording performance of the BD-R with this Ge/GeCu bilayer were investigated in detail. Additionally, a BD-R with a Ge_67_Cu_33_ (16 nm) layer was also prepared as a contrasting sample.

## 2. Experimental Procedures 

The Ge_67_Cu_33_ (16 nm) and the Ge (3 nm)/Ge_67_Cu_33_ (16 nm) films were deposited on naturally oxidized Si, glass, and polycarbonate (PC) substrates at room temperature by sputtering. For the films’ growth, Ge and GeCu targets were used. To avoid oxidation in these two recording layers, they were sandwiched with ZnS–SiO_2_ protective layers. When the base pressure was lower than 5 × 10^−7^ Torr, the Ar gas was introduced into the deposition chamber, and the working pressure of film growth was kept at 5 mTorr. In addition, to investigate the crystallization mechanism of these recording films, some samples were subjected to an annealing processes in a furnace under a vacuum environment for 15 min and then quenched in ice water. 

**Figure 1:**
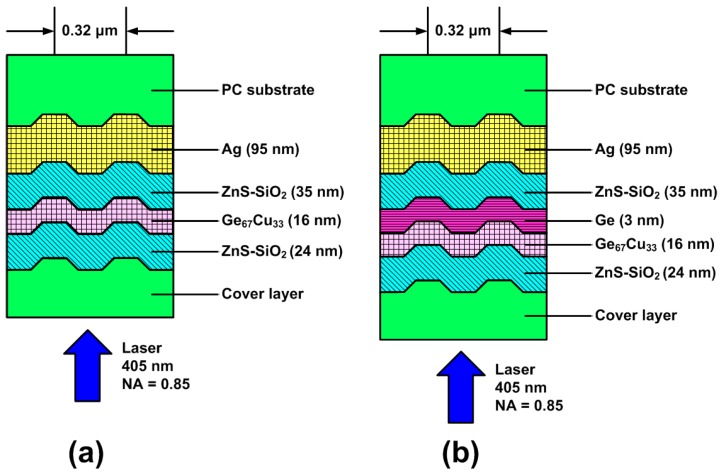
Layer structures of the write-once blu-ray discs (BD-Rs) with (**a**) Ge_67_Cu_33_ (16 nm) and (**b**) Ge (3 nm)/Ge_67_Cu_33_ (16 nm) recording films. PC: polycarbonate.

For the sake of dynamic tests, the on-groove recording method was adopted. Thus, a 1.1-mm-thick PC substrate with a 0.32 μm track pitch was employed for the preparation of a BD-R. For the deposition of the layer structure of the BD-R, an Ag (95 nm) reflective layer, an upper dielectric layer of ZnS-SiO_2_ (35 nm), a recording layer, and a lower dielectric layer of ZnS-SiO_2_ (24 nm) were deposited on the PC substrate in sequence. Finally, a 0.1-mm-thick transparent PC cover layer was prepared on the top of these layers via the spin-coating method. The layer structures of BD-Rs with the Ge_67_Cu_33_ (16 nm) and the Ge (3 nm)/Ge_67_Cu_33_ (16 nm) recording films are depicted in Figure 1.

Thermal properties of the Ge_67_Cu_33_ (16 nm) and the Ge (3 nm)/Ge_67_Cu_33_ (16 nm) films were investigated by a home-made transient reflectivity-temperature measurement system from room temperature to 500 °C. The sample was mounted on a Linkam THMS 600 heating stage (Linkam, Surrey, UK), and the measurement was performed in an argon protective atmosphere. A blue laser with a wavelength of 405 nm was used in the measurement, and the reflectivity variation with increasing temperature could be recorded in real time during the heating process. Microstructures and crystallization mechanisms of these two films were determined by transmission electron microscopy (TEM) (JEM2100F, JEOL, Tokyo, Japan). Relationships between optical reflectivity and wavelength were analyzed via a dual-beam UV–VIS–NIR spectrophotometer (Lambda 900, Perkin Elmer, Waltham, MA, USA) with a 150-mm Spectralon-coated integrating sphere. The integrating sphere was employed to collect the reflected light from the sample over a full hemisphere. The detection was performed by a photomultiplier (Perkin Elmer) located at the bottom of the integrating sphere. The composition of the GeCu alloy film was measured by an electron probe micro-analyzer. Jitter values and modulations as a function of writing power for the BD-Rs were characterized by a dynamic tester (ODU-1000, PULSTEC, Shizuoka, Japan). In the dynamic test, a wavelength of 405 nm and an objective lens with a numerical aperture (NA) of 0.85 were used. The modulation code is (1, 7) RLL. Additionally, the linear velocities of 2× and 4× recording speeds are 9.84 and 19.68 m/s, respectively.

## 3. Results and Discussion

**Figure 2:**
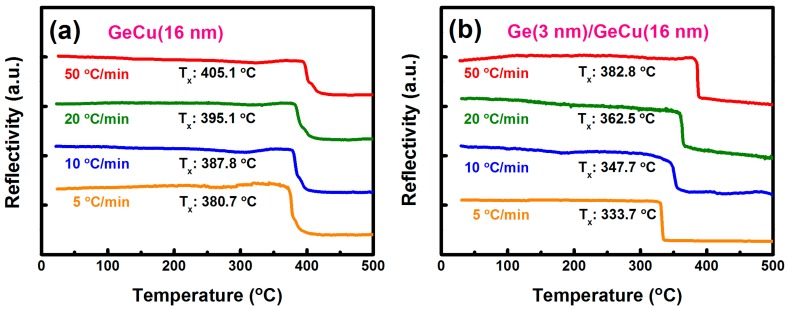
Relationships between reflectivity and temperature of (**a**) Ge_67_Cu_33_ (16 nm) and (**b**) Ge (3 nm)/Ge_67_Cu_33_ (16 nm) films measured at the heating rates of 5, 10, 20, and 50 °C/min.

**Figure 3:**
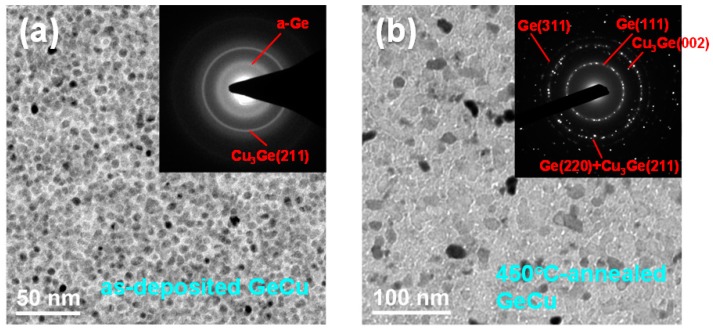
Transmission electron microscopy (TEM) bright field images and electron diffraction patterns of (**a**) as-deposited and (**b**) 450 °C-annealed Ge_67_Cu_33_ (16 nm) films.

**Figure 4:**
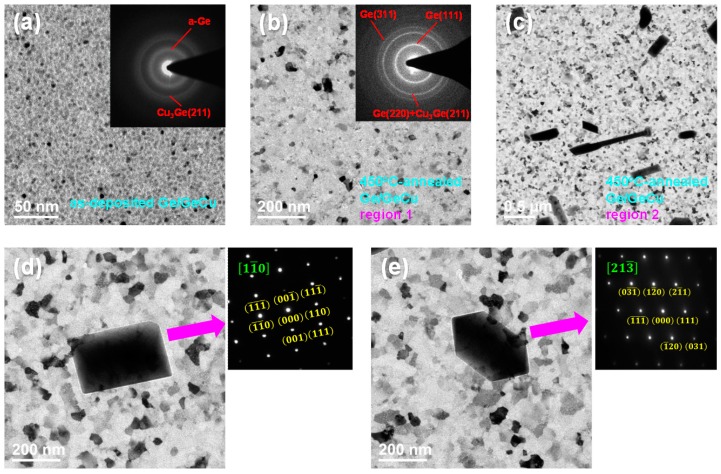
(**a**) TEM image and electron diffraction pattern of as-deposited Ge (3 nm)/Ge_67_Cu_33_ (16 nm) film; (**b**) TEM image and electron diffraction pattern of annealed Ge/GeCu film; (**c**) TEM image of region 2 of the annealed film; (**d**) TEM bright field image and selected area electron diffraction pattern of parallelogram-shaped grain formed in the 450 °C-annealed Ge/GeCu film; (**e**) TEM bright field image and selected area electron diffraction pattern of hexagon-shaped grain formed in the 450 °C-annealed Ge/GeCu film.

Figure 2 shows the relationships between the optical reflectivity and temperature of the Ge_67_Cu_33_ (16 nm) and the Ge (3 nm)/Ge_67_Cu_33_ (16 nm) films. The reflectivity–temperature measurements of these two films were performed at the heating rates of 5, 10, 20, and 50 °C/min. As shown in Figure 2a, the GeCu film presents an abrupt decrease in the reflectivity between 372.3 and 419.7 °C. Besides, the Ge (3 nm)/Ge_67_Cu_33_ (16 nm) bilayer also exhibits a similar reduction in the reflectivity between 329.4 and 387.5 °C, as displayed in Figure 2b. We can observe that the curves shown in Figure 2 are vertically shifted. Generally, the variation in the reflectivity reveals that the film undergoes a structural change, which can modify its optical characteristics [13]. Based on the TEM observations (as discussed later in Figure 3 and Figure 4), the changes of reflectivity for the Ge_67_Cu_33_ (16 nm) and the Ge (3 nm)/Ge_67_Cu_33_ (16 nm) films are both attributed to the crystallization of a-Ge. The crystallization temperatures (*T_x_*) of these two films can be defined as the temperature at a midpoint of the reflectivity variation. The crystallization temperatures of the Ge_67_Cu_33_ (16 nm) film measured at the heating rates of 5, 10, 20, and 50 °C/min were 380.7, 387.8, 395.1, and 405.1 °C, respectively. Meanwhile, the crystallization temperatures of the Ge (3 nm)/Ge_67_Cu_33_ (16 nm) bilayer at these four heating rates were determined to be 333.7, 347.7, 362.5, and 382.8 °C, respectively. Apparently, in comparison to the Ge_67_Cu_33_ (16 nm) film, the crystallization temperature of a-Ge can be further decreased by adding a 3-nm-thick Ge layer.

Figure 3 shows the TEM bright field images and electron diffraction patterns of the as-deposited and the 450 °C-annealed Ge_67_Cu_33_ (16 nm) films. Uniform grains of size 3 nm–5 nm were found in the as-deposited Ge_67_Cu_33_ (16 nm) film, as displayed in Figure 3a. Moreover, its electron diffraction pattern is identified as a Cu_3_Ge(211) phase. After annealing at 450 °C, the grain size of this film increased to 10 nm–30 nm, as shown in Figure 3b. The electron diffraction pattern of the annealed GeCu film can be identified as Cu_3_Ge and cubic Ge phases.

Figure 4 exhibits TEM bright field images and electron diffraction patterns of the as-deposited and the 450 °C-annealed Ge (3 nm)/Ge_67_Cu_33_ (16 nm) films. As shown in Figure 4a, we can observe that the microstructure and the crystalline phase of the as-deposited Ge/GeCu film are both similar to those of the as-deposited GeCu film (Figure 3a). Figure 4b shows one microstructural feature of the 450 °C-annealed Ge/GeCu film, which is defined as region 1. On the other hand, another feature (defined as region 2) can also be found in the TEM image of the 450 °C-annealed Ge/GeCu film, as shown in Figure 4c. In Figure 4b, after annealing the Ge/GeCu film at 450 °C for 15 min, the grain size of this film increased to 10 nm–70 nm. The electron diffraction pattern of Figure 4b can be determined to be Cu_3_Ge and cubic Ge phases. Based on the above observations, after the annealing process, the increment of grain size and the formation of a cubic Ge phase both appeared in the GeCu and the Ge/GeCu films, as compared with their as-deposited films. Furthermore, the annealed Ge/GeCu film had a larger grain size and more obvious diffraction rings than those of the annealed GeCu film. This indicates that the phenomenon of Ge crystallization is much more apparent in the annealed Ge/GeCu film. In other words, the addition of a 3-nm-thick Ge layer is helpful for the crystallization of a-Ge in the Ge/GeCu film. According to the reflectivity–temperature results (Figure 2), the reflectivities of GeCu and Ge/GeCu films reduced upon raising the measurement temperature from 372.3 to 419.7 °C and from 329.4 to 387.5 °C, respectively, which can both be attributed to the formation of Ge crystallization. In Figure 4c, there were several extremely large grains (300 nm–1200 nm) formed in the 450 °C-annealed Ge/GeCu film. Some of these large grains possessed unique geometrical shapes consisting of rods, rectangles, parallelograms, hexagons, and so on. To determine the crystalline phase of these large grains, we selected two grains and examined them via the selected area electron diffraction method. Then, the structural information was calculated using the crystal structure theory. As shown in Figure 4d, the phase of the parallelogram-shaped grain was cubic Ge with a $\left[1\bar{1}0\right]$ zone axis. Meanwhile, the hexagon-shaped grain was also identified as cubic Ge phase, and it had a $\left[21\bar{3}\right]$ zone axis (Figure 4e). It can be reasonably suggested that the large grains formed in the 450 °C-annealed Ge/GeCu film all belonged to a cubic Ge phase. It is worth mentioning that these large grains with unique geometrical shapes do not appear in the Ge/Au [7], Ni/Ge [8], GeCu [9], Ge/Cu [10], NiGe [11], or Ge/NiGe [12] films, implying that the layered structure of Ge/GeCu is beneficial for the formation of these grains.

**Figure 5:**
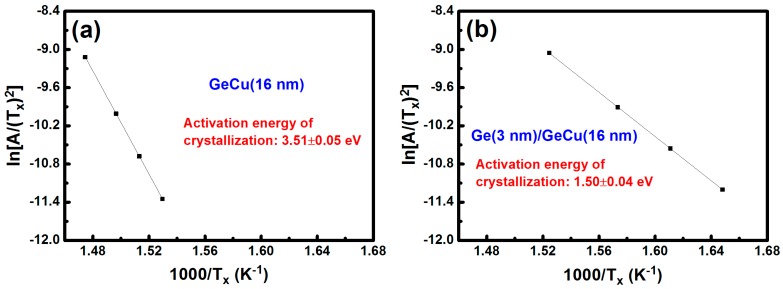
Plots of ln[*A*/(*T_x_*)^2^] versus (1/*T_x_*) of (**a**) Ge_67_Cu_33_ (16 nm) and (**b**) Ge (3 nm)/Ge_67_Cu_33_ (16 nm) films for the Ge crystallization.

Based on the TEM results, it can be seen that the change in reflectivity with increasing temperature (Figure 2) for these two films is ascribed to the formation of Ge crystallization. To estimate the crystallization speed of Ge in these two films, Kissinger’s method is used [14], and the crystallization temperatures obtained at various heating rates (discussed in Figure 2) are required for this method. These temperatures determined at various heating rates were substituted into the Kissinger’s equation [14] to evaluate the activation energy. According to the Kissinger’s equation:

ln[*A*/(*T_x_*)^2^] = (−*E_a_*/*K_b_*) × (1/*T_x_*) + const. (1)

where *A* is the heating rate, *T_x_* is the crystallization temperature, *K_b_* is the Boltzmann constant (8.6 × 10^−5^ eV/K), and *E_a_* is the activation energy. The activation energy can be estimated via a line slope of the ln[*A*/(*T_x_*)^2^] versus (1/*T_x_*) curve. The plots of ln[*A*/(*T_x_*)^2^] versus (1/*T_x_*) of the Ge_67_Cu_33_ (16 nm) and the Ge (3 nm)/Ge_67_Cu_33_ (16 nm) films for the crystallization of Ge are shown in Figure 5a,b, respectively. After linear fitting to the data, the activation energies of Ge crystallization were calculated to be 3.51 ± 0.05 eV and 1.50 ± 0.04 eV for the GeCu and the Ge/GeCu films, respectively. Apparently, the activation energy of Ge crystallization for the Ge/GeCu bilayer was much lower than that of the GeCu film, revealing that the Ge/GeCu bilayer had a higher crystallization speed. In other words, the Ge/GeCu bilayer is more suitable for high-speed optical recording applications than the GeCu film. Although the activation energy of 1.50 ± 0.04 eV for the Ge/GeCu bilayer is relatively low, it is still thermally stable enough for optical recording media. Because of the significant decrease in the activation energy of Ge crystallization (i.e., the increment of crystallization speed), the grain size of the annealed Ge/GeCu bilayer is larger than that of the annealed GeCu film, as shown in Figure 3 and Figure 4. 

**Figure 6:**
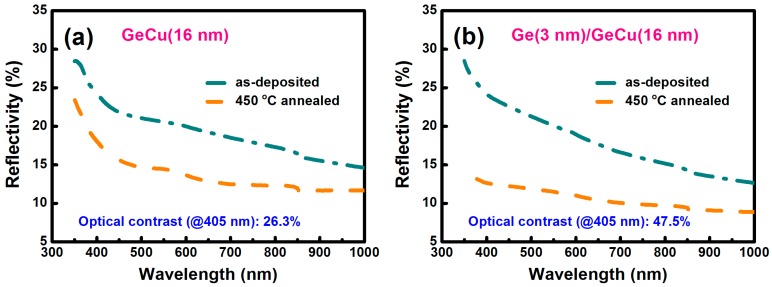
Reflectivity spectra of the as-deposited and the 450 °C-annealed states for (**a**) Ge_67_Cu_33_ (16 nm) and (**b**) Ge (3 nm)/Ge_67_Cu_33_ (16 nm) films.

Figure 6 shows the reflectivity spectra of the as-deposited and the 450 °C-annealed states for the Ge_67_Cu_33_ (16 nm) and the Ge (3 nm)/Ge_67_Cu_33_ (16 nm) films. The measured wavelength was increased from 350 to 1000 nm. For the as-deposited state, the reflectivities (@ 405 nm) of the GeCu and the Ge/GeCu films were 23.9% and 24.0%, respectively. In addition, the reflectivities (@ 405 nm) of these two 450 °C-annealed films were measured to be 17.6% and 12.6%, respectively. The optical contrast is defined as ((R_1_ − R_2_)/R_1_) × 100%, where R_1_ and R_2_ are the reflectivities of the as-deposited and the 450 °C-annealed states, respectively. For the GeCu and the Ge/GeCu films, the optical contrasts (@ 405 nm) were determined to be 26.3% and 47.5%, respectively. From the TEM results (Figure 4), the more significant reduction in the reflectivity of the Ge/GeCu film could be owing to the formation of the more obvious Ge crystallization, which is helpful to enhance the optical contrast. This indicates that the Ge/GeCu bilayer has more potential than the GeCu film for application in blue laser recording media.

**Figure 7:**
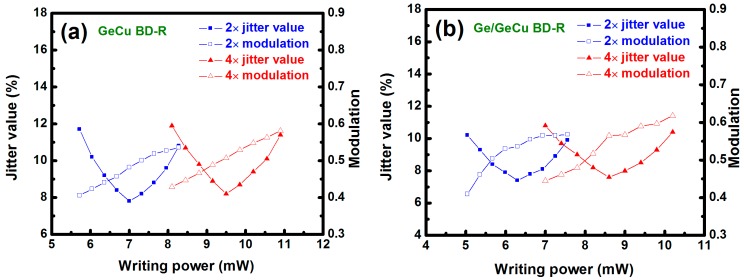
Jitter values and modulations as a function of writing power at 2× and 4× recording speeds for the BD-Rs with (**a**) Ge_67_Cu_33_ (16 nm) and (**b**) Ge (3 nm)/Ge_67_Cu_33_ (16 nm) recording films.

For the dynamic tests, the Ge_67_Cu_33_ (16 nm) and the Ge (3 nm)/Ge_67_Cu_33_ (16 nm) films were both selected as the recording layers to fabricate the BD-Rs. The results of dynamic tests for these two BD-Rs are displayed in Figure 7. In the dynamic tests, the jitter value and the modulation were measured with varying writing power at 2× and 4× recording speeds. Generally, a low jitter value indicates a lower error as the marks are written in the disc. It was found that the measured modulations at various writing powers and recording speeds for these two BD-Rs were all higher than 0.4. For the BD-R with the GeCu recording film, the optimum jitter values were 7.8% at 7 mW and 8.2% at 9.5 mW, respectively, for 2× and 4× recording speeds (Figure 7a). On the other hand, when the Ge/GeCu bilayer was used as the BD-R recording film, these two optimum jitter values were 7.4% at 6.3 mW and 7.6% at 8.6 mW, respectively, as shown in Figure 7b. We can observe that the optimum jitter value of the BD-R with the Ge/GeCu recording film was obtained at the lower writing power in comparison to that with the GeCu recording film, resulting from the lower crystallization temperature of the Ge/GeCu bilayer. The results demonstrate that the Ge (3 nm)/Ge_67_Cu_33_ (16 nm) recording film is highly feasible for BD-R applications.

## 4. Conclusions 

In summary, the thermal characteristics, crystallization mechanisms, and optical properties of Ge_67_Cu_33_ (16 nm) and Ge (3 nm)/Ge_67_Cu_33_ (16 nm) films have been analyzed in detail. In addition, the recording performances of the BD-Rs using these two recording films were also investigated. The results indicate that the Ge/GeCu recording film is much more feasible for BD-R applications than the GeCu recording film. The crystallization temperature of a-Ge for the Ge/GeCu bilayer is as low as 333.7 °C–382.8 °C. After annealing at 450 °C, the formation of Ge crystallization in the Ge/GeCu bilayer is quite obvious, which is helpful to increase the optical contrast of this film. The dynamic tests showed that the optimum jitter values of the BD-R using the Ge/GeCu recording film were 7.4% at 6.3 mW and 7.6% at 8.6 mW, respectively, for 2× and 4× recording speeds.
